# Accounting for diet and age

**DOI:** 10.7554/eLife.80890

**Published:** 2022-07-15

**Authors:** Hélène Tonnelé, Amelie Baud

**Affiliations:** 1 Centre for Genomic Regulation, Barcelona Institute of Science and Technology Barcelona Spain; 2 Universitat Pompeu Fabra Barcelona Spain

**Keywords:** heritability, gene-environment interaction, longitudinal, quantitative trait locus, mixed models, diversity outbred mice, Mouse

## Abstract

The diet and age of mice can modulate how different genetic variants impact body weight, demonstrating the need to take context into account when performing genetic studies.

**Related research article** Wright KM, Deighan AG, Di Francesco A, Freund A, Jojic V, Churchill GA, Raj A. 2022. Age and diet shape the genetic architecture of body weight in diversity outbred mice. eLife **11**:e64329. doi: 10.7554/elife.64329.

The human genome is made up of 3 billion base pairs, but variations in just 1% of them cause differences in traits observed between individuals, from blood cell counts to body weight and behaviour (Walters et al., 2019). This 1% is extremely important to geneticists for several reasons (Timpson et al., 2018). First, they want to understand how much genetics contributes to differences between individuals. Second, knowing more about this 1% of the genome could help doctors predict whether a person is likely to develop certain diseases. Finally, identifying the genetic variants that affect a specific trait can be the first step towards unravelling its underlying biological mechanism. For traits related to disease, this can then result in the development of therapeutic interventions.

Determining how genetic variants lead to a specific phenotype is a real challenge. Most common traits and diseases – including type 2 diabetes and major depression – are affected by thousands of genetic variants across the genome, each with a tiny effect on the trait studied (Timpson et al., 2018). Now, in eLife, Anil Raj (Calico Life Sciences), Gary Churchill (Jackson Laboratory) and colleagues – including Kevin Wright as first author – report that the effect of a genetic variant on the body weight of mice can be diet- and age-dependent, further complicating the relationship between genes and traits (Wright et al., 2022).

Body weight is a quantitative trait that can easily be measured in hundreds of mice, making it easier to detect subtle changes. It also lends itself to studying age-related effects, since mice can be weighed each week of their lives without the procedure affecting the animal. To investigate these effects, Wright et al. weighed 960 female mice once per week between the ages of 60 and 660 days old. The mice were given unlimited food (ad libitum feeding) until they were 180 days old, and were then randomly assigned to one of five dietary groups: ad libitum feeding, 20% caloric restriction, 40% caloric restriction, fast one day per week, or fast two days per week.

Each mouse was also genotyped to ascertain the two letters of its DNA at each genomic position known to vary in this mouse population. With these data in hand, Wright et al. proceeded to dissect the genetic architecture of body weight in mice, yielding one of the finest studies in the field. Using software developed specifically to capture context-dependent genetic effects, such as age and diet (Dahl et al., 2020), Wright et al. found that before dietary intervention at 180 days of age, genetics made a substantial contribution to variation in body weight, accounting for 60% to 80% of the weight variance observed in the mice. After dietary intervention, however, diet became the most important factor explaining variation in body weight. In the group with a 40% caloric restriction, genetics kept explaining about 80% of the weight variance, but in the other dietary groups, genetic effects decreased over time.

Wright et al. next sought to identify individual genetic loci influencing body weight and to investigate the relationship between genetic effects and diet. To do so, they leveraged the fact that the mice they studied are all descended from eight known founders many generations ago (Svenson et al., 2012) and that these founders differed from each other at 34.5 million base-pairs across the genome. In the mouse population studied by Wright et al., each chromosome is a fine-grained mosaic of the eight founder genomes and, at each locus, the eight ancestral haplotypes (small chromosome chunks inherited from the founders) can be recognised using appropriate software (Broman et al., 2019). To identify loci influencing body weight, the mice can be divided based on the haplotype they have at the locus and, if there are differences in body weight between the haplotype groups, this is evidence for ‘diet-independent’ effects. The mice can also be divided based on both their dietary group and their ancestral haplotype; in this case, variations between the haplotype groups that differ in magnitude between the dietary groups point to ‘diet-dependent’ effects (Figure 1).

**Figure 1.**
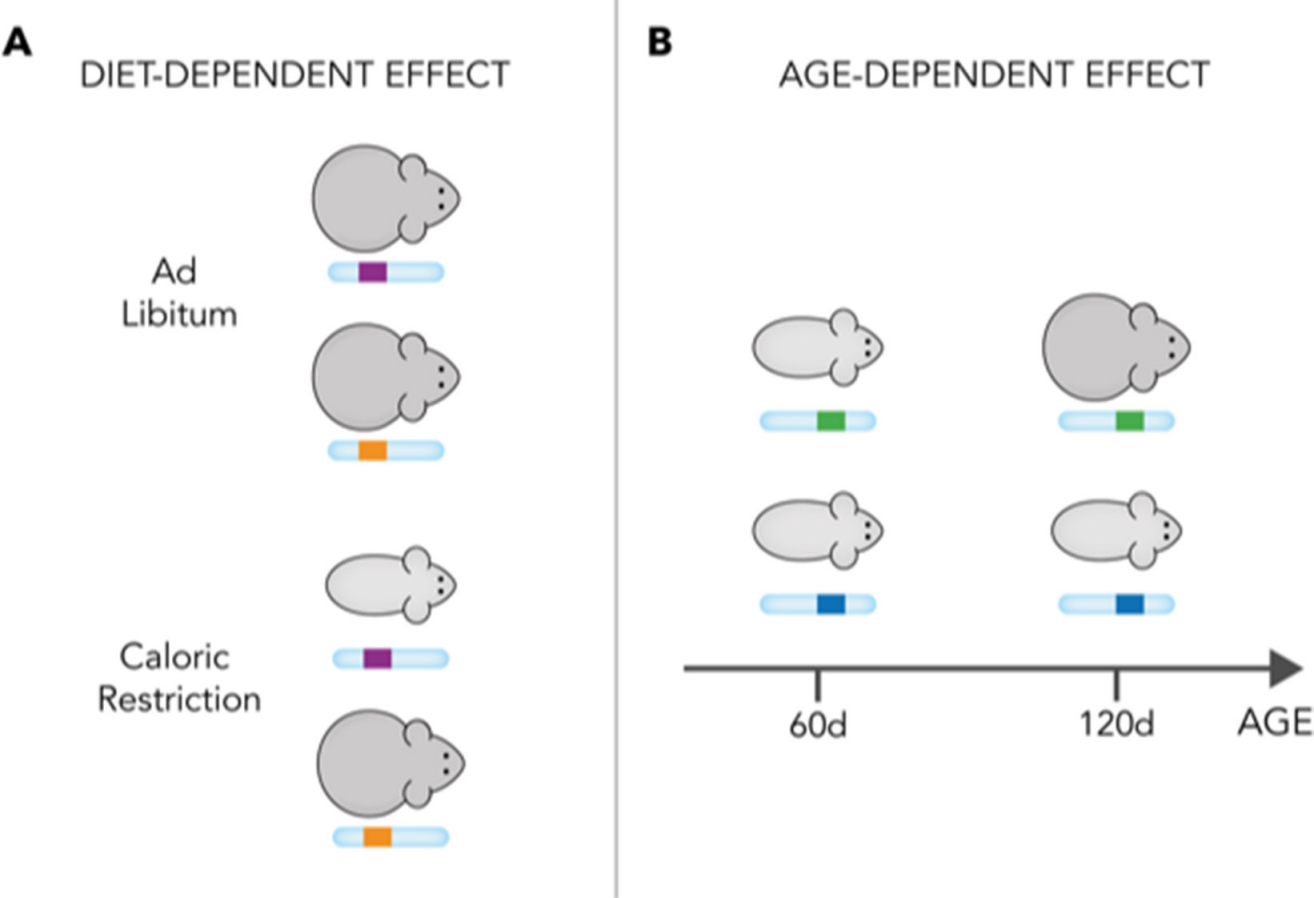
Schematic representation of diet- and age-dependent genetic effects. The effect of founder haplotypes on body weight can be diet-dependent (**A**) or age-dependent (**B**). In this figure, for simplicity, there are two haplotypes at each locus: purple (upper) and orange (lower) for the variant in (**A**); and green (upper) and blue (lower) for the variant in (**B**). In reality, however, there are eight different haplotypes at each locus in the mice studied by Wright et al. The haplotypes in (**A**) have an effect on body weight only when the mice are fed a caloric-restricted diet (bottom). In this situation, the purple haplotype leads to lower weight. This indicates that the haplotypes have a diet-dependent effect. Similarly, the haplotypes in (**B**) have an effect on body weight only after 120 days of age, when the green haplotype (top) causes increased body weight.

Wright et al. identified five loci with only diet-independent effects and ten with only diet-dependent effects. This finding demonstrates that failing to model diet-dependent effects will result in missing genetic loci important to determining body weight. Additionally, Wright et al. also identified nine loci with both diet-independent and diet-dependent effects, raising the question of whether the same haplotype groupings give rise to the two types of effects. They found that different haplotype groupings give rise to diet-independent and diet-dependent effects in all cases. This supports the notion that different genetic effects drive diet-independent differences in body weight across the entire population and diet-dependent differences within dietary groups. In terms of the specific DNA code, this means that these loci harbour multiple base-pair variants and that different variants or different combinations of letters at the same variants give rise to diet-independent and diet-dependent effects.

In addition to identifying diet-dependent effects, Wright et al. also demonstrate that genetic variants can have different effects at different ages, and that this trend can be non-linear. For example, the effect of a specific genetic variant could increase, peak at a certain age, and then decrease. A future study on the same mice will examine the interplay between genetics and diet in the context of longevity.

Wright et al.’s findings highlight the need for taking context into account in genetic studies. This need has been recognised for some time, but it is difficult to meet due to uncertainty about what the relevant context might be for any given trait (Ashbrook et al., 2021; McAllister et al., 2017). Moreover, the statistical models needed to account for context are getting more and more complex, requiring ever larger sample sizes. With their results, Wright et al. make significant headway in this field.
